# Respiratory exchange ratio overshoot during exercise recovery: a promising prognostic marker in HFrEF

**DOI:** 10.1007/s00392-024-02391-9

**Published:** 2024-02-15

**Authors:** Marco Vecchiato, Daniel Neunhaeuserer, Emanuele Zanardo, Giulia Quinto, Francesca Battista, Andrea Aghi, Stefano Palermi, Luciano Babuin, Chiara Tessari, Marco Guazzi, Andrea Gasperetti, Andrea Ermolao

**Affiliations:** 1 Sports and Exercise Medicine Division, Department of Medicine, University of Padova, University Hospital of Padova, Via Giustiniani 2, 35128 Padova, Italy; 2 Fisioterapia Osteopatia Raimondi Di Giovanni e Daniele, Piazza Vittorio Veneto 1, Selvazzano Dentro, Padova, Italy; 3 Public Health Department, University of Naples Federico II, 80131 Naples, Italy; 4 Cardiology Unit, Department of Cardiac, Thoracic, Vascular Sciences and Public Health, University Hospital of Padova, Padova, Italy; 5 Cardiac Surgery Unit, Department of Cardiac, Thoracic, Vascular Sciences and Public Health, University Hospital of Padova, Padova, Italy; 6 Department of Biological Sciences, University of Milano School of Medicine, Milan, Italy; 7 Cardiology Division, San Paolo Hospital, Milan, Italy

**Keywords:** RER, Cardiopulmonary exercise testing, CPET, Recovery kinetics, MACEs, Survival

## Abstract

**Background and aims:**

Transient increases (overshoot) in respiratory gas analyses have been observed during exercise recovery, but their clinical significance is not clearly understood. An overshoot phenomenon of the respiratory exchange ratio (RER) is commonly observed during recovery from maximal cardiopulmonary exercise testing (CPET), but it has been found reduced in patients with heart failure with reduced ejection fraction (HFrEF). The aim of the study was to analyze the clinical significance of these RER recovery parameters and to understand if these may improve the risk stratification of patients with HFrEF.

**Methods:**

This cross-sectional study includes HFrEF patients who underwent functional evaluation with maximal CPET for the heart transplant checklist at our Sports and Exercise Medicine Division. RER recovery parameters, including RER overshoot as the percentual increase of RER during recovery (RER mag), have been evaluated after CPET with assessment of hard clinical long-term endpoints (MACEs/deaths and transplant/LVAD-free survival).

**Results:**

A total of 190 patients with HFrEF and 103 controls were included (54.6 ± 11.9 years; 73% male). RER recovery parameters were significantly lower in patients with HFrEF compared to healthy subjects (RER mag 24.8 ± 14.5% vs 31.4 ± 13.0%), and they showed significant correlations with prognostically relevant CPET parameters. Thirty-three patients with HFrEF did not present a RER overshoot, showing worse cardiorespiratory fitness and efficiency when compared with those patients who showed a detectable overshoot (VO_2_ peak: 11.0 ± 3.1 vs 15.9 ± 5.1 ml/kg/min; VE/VCO_2_ slope: 41.5 ± 8.7 vs 32.9 ± 7.9; ΔPETCO_2_: 2.75 ± 1.83 vs 4.45 ± 2.69 mmHg, respectively). The presence of RER overshoot was associated with a lower risk of cardiovascular events and longer transplant-free survival.

**Conclusion:**

RER overshoot represents a meaningful cardiorespiratory index to monitor during exercise gas exchange evaluation; it is an easily detectable parameter that could support clinicians to comprehensively interpreting patients’ functional impairment and prognosis. CPET recovery analyses should be implemented in the clinical decision-making of advanced HF.

**Graphical Abstract:**

**RER Overshoot during CPET recovery phase in HFrEF**

Transient increases, also called overshoot, in respiratory exchange ratio (RER) have been observed during exercise recovery in healthy subjects and patients with chronic diseases. A total of 190 patients with HFrEF who underwent CPET for heart transplant checklist were analyzed and compared with 103 controls, using a protocol to monitor gas exchange during recovery phase. RER overshoot was significantly lower in patients with HFrEF than controls, and some patients with HFrEF (17.4%) presented no overshoot. The presence of RER overshoot was associated with higher aerobic capacity and cardiorespiratory efficiency with lower risk of cardiovascular events and longer transplant/LVAD-free survival. HFrEF, heart failure with reduced ejection fraction; CPET, cardiopulmonary exercise testing; LVAD, left ventricular ejection fraction.

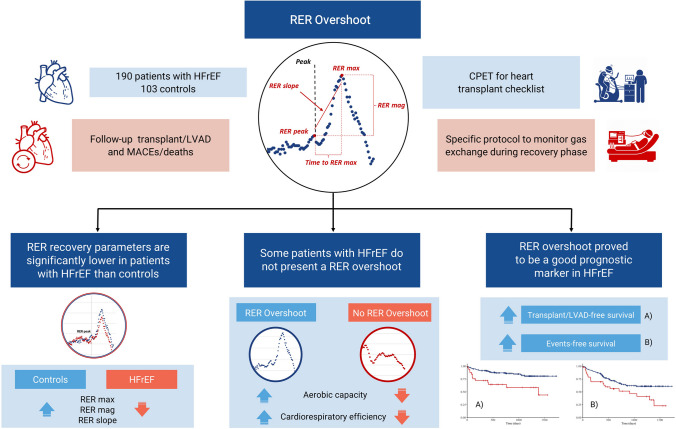

**Supplementary Information:**

The online version contains supplementary material available at 10.1007/s00392-024-02391-9.

## Introduction

Cardiopulmonary exercise testing (CPET) is recognized as the gold standard in the evaluation of physical fitness, efficiency and exercise limitations in athletes, healthy subjects and patients with different chronic diseases [1]. CPET is routinely used in the prognostic evaluation of patients with heart failure with reduced ejection fraction (HFrEF) in whom its prognostic value is powerful and well-established [2, 3].

Among the several proposed CPET parameters, some have been shown to be important prognostic markers for various diseases, including patients with HFrEF, who can be affected by different cardiorespiratory limitations to exercise [4]. The peak oxygen uptake (VO_2_) is strongly influenced by maximal cardiac output during exercise and is thus frequently used for clinical decision-making in the evaluation of heart transplant candidates in HFrEF [3, 5].

However, whereas many studies have focused on the cardiopulmonary response at rest and during exercise, the recovery phase post-maximal exercise has not been studied yet and lacks a routine application in daily practice.

Recently, the overshoot phenomenon of some CPET-derived variables during the recovery phase has been investigated, particularly the respiratory exchange ratio (RER) [6]. The overshoot magnitude of this parameter is higher in healthy subjects compared to patients with HFrEF, but its correlation with the degree of cardiac impairment in resting conditions remains undefined [6, 7].

According to these premises and considering that scientific evidence on overshoot physiology and clinical impact is still limited, the aim of the present study was to evaluate the RER recovery indices in a population with HFrEF to understand whether these CPET parameters could help in the prognostic evaluation of certain subpopulations of patients.

## Methods

### Study and patients

This was a cross-sectional study including all HFrEF patients who underwent functional evaluation at the Sports and Exercise Medicine Division of the University Hospital of Padova between January 2018 and December 2021 for the heart transplant checklist. The exclusion criteria were left ventricular ejection fraction (LVEF) > 40%, the presence of a left ventricular assist device (LVAD) at first evaluation, being classified as NYHA Class IV, and contraindications to performing the CPET (e.g., severe orthopedic conditions or recent thoracic and/or abdominal surgery). Patients with less than 4 min of monitored gas exchange during recovery were also excluded. Because of the novelty and relatively poor description in the literature of the phenomenon investigated, in addition to the cohort of patients with HFrEF, a group of healthy subjects without structural or functional heart disease was selected. All patients provided written informed consent before functional evaluation. The investigation conforms with the principles outlined in the Declaration of Helsinki, and the study was approved by the local Ethics Committee of the University Hospital of Padova (protocol n. 302n/AO/22—date: 13.10.2022).

### Exercise testing protocol

Maximal CPET (Jaeger Masterscreen-CPX, Carefusion) with an incremental ramp protocol of 5/10 W × min for patients with HFrEF and 20/25 W × min for controls aiming to reach exhaustion within 8 to 12 min was performed on a cycle-ergometer (eBike, GE Healthcare) until patients reached a Borg rating of perceived exertion (RPE) ≥ 18/20. Continuous monitoring of the electrocardiogram was performed throughout the test, and the respiratory gas exchange (VO_2_, VCO_2_) and ventilation (VE) were monitored breath by breath during the whole test (data averaged for every 20 s) and at least the first 4 min of recovery. The patients followed a cool down phase for about 3 min post-exercise after which they were in a lying position for the rest of the recovery phase. VO_2_ peak was defined as the highest value of VO_2_ attained in a 30-s interval. The ventilatory threshold (VT) was identified on the plots of the cardiopulmonary evaluation using the simplified V-Slope method [8]. The minute ventilation/carbon dioxide production (VE/VCO_2_) slope was calculated as the coefficient of linear regression from the beginning of the exercise test (excluding initial hyperventilation) to the respiratory compensation point (RCP) [9]. Partial pressure end-tidal carbon dioxide (PETCO_2_) was measured at rest and during exercise. The difference between the resting (PETCO_2_ rest) and maximum value during exercise (PETCO_2_ max) was indicated as ΔPETCO_2_. Exercise oscillatory ventilation (EOV) was defined as oscillations in VE with an amplitude > 15% of resting VE and duration > 60% of exercise duration [10]. The VO_2_/work rate slope was calculated as the linear regression coefficient of the entire exercise phase. The oxygen uptake efficiency slope (OUES) was calculated as the coefficient of the linear relationship between VO_2_ and the logarithm of total ventilation [11].

### Overshoot analyses

**Fig. 1:**
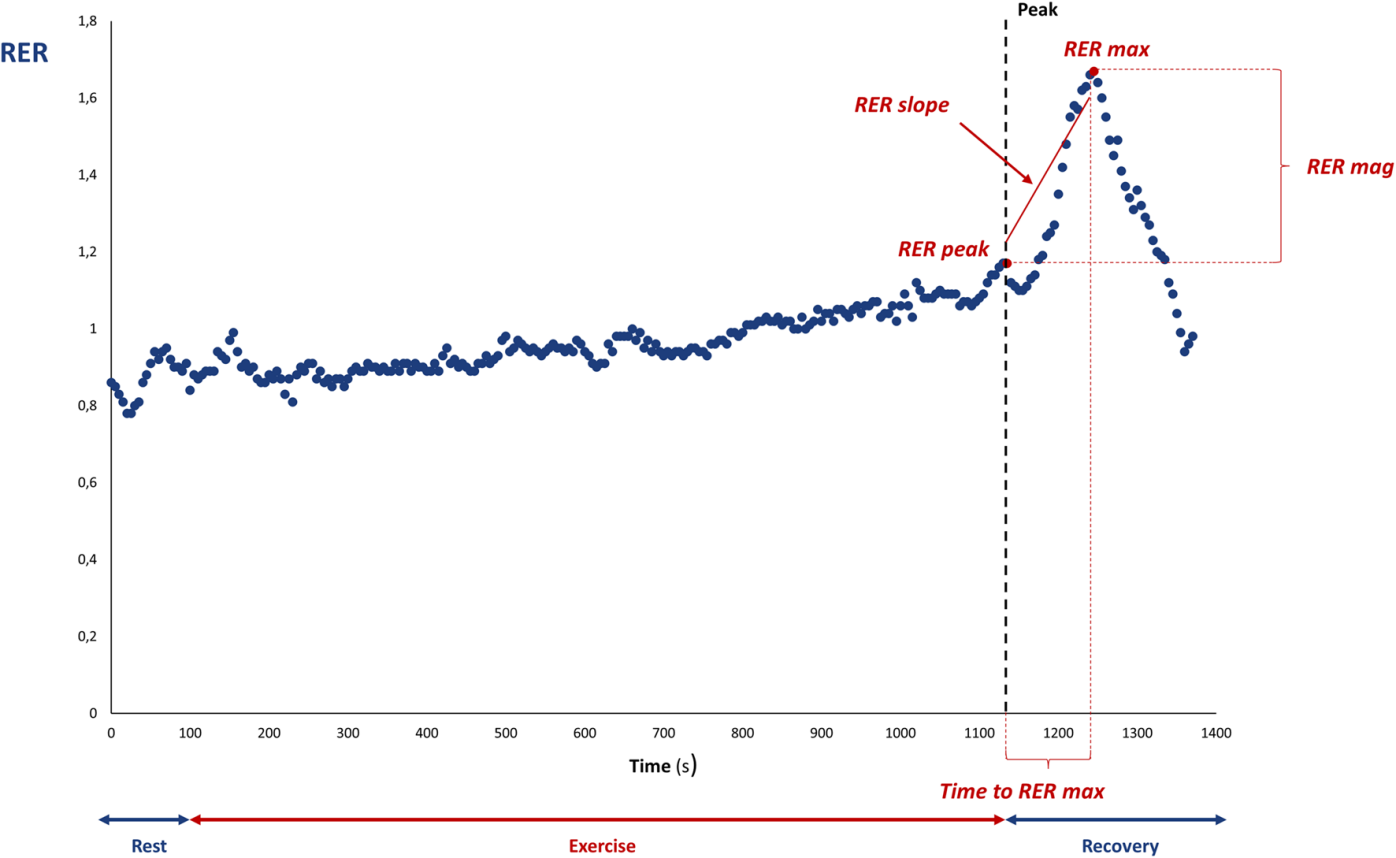
The RER overshoot. An example of the recovery of the respiratory exchange ratio (RER) in a healthy subject. aRER peak is the RER value recorded at peak exercise intensity. RER max is the highest RER value recorded during recovery. Time to RER max was defined as the time (in seconds) needed to reach RER max during recovery. RER mag was defined as the percentual increase in the RER during recovery compared with RER peak. RER slope is the linear regression slope of the RER increase after the end of exercise

The behavior of the RER during recovery was analyzed by assessing five parameters: RER at peak exercise (RER peak), the maximum RER value reached during recovery (RER max), the magnitude of the RER overshoot as the percentual increase of RER during recovery (RER mag), the time needed from RER peak to RER max (time to RER max), and the linear regression slope of the RER increase after the end of exercise (RER slope) [6]. Figure 1 describes how peak RER, RER max, RER mag, and time to RER max have been evaluated. The magnitudes of the VE/VO_2_ and PETO_2_ overshoots were calculated with the same modality.

### Subgroup analysis

Patients with HFrEF were further subclassified to investigate the behavior of the RER in the various subgroups with potential functional and prognostic differences. Firstly, patients were divided based on the VE/VCO_2_ slope into ventilatory classes: ventilatory class I with VE/VCO_2_ slope < 30; ventilatory class II with VE/VCO_2_ slope between 30 and 35.9; ventilatory class III with VE/VCO_2_ slope between 36 and 44.9; ventilatory class IV with VE/VCO_2_ slope ≥ 45. Furthermore, patients were grouped based on Weber’s classes: Class A with VO_2_ peak ≥ 20 ml/kg/min; Class B with VO_2_ peak between 16 and 19.9 ml/kg/min; Class C with VO_2_ peak between 10 and 15.9 ml/kg/min; Class D with VO_2_ peak < 10 ml/kg/min [4]. Finally, patients were grouped based on the presence or absence of a RER overshoot during recovery from CPET.

### Statistical analyses

Statistical analyses were performed using IBM SPSS Statics software version 26, and normality distributions were assessed using the Shapiro–Wilk test. Data are expressed as a mean ± the standard deviation. The difference between subgroups was assessed with an unpaired *t*-test for the normally distributed variables and a Mann–Whitney U test for the non-normally distributed variables. The correlations were evaluated with Pearson’s or Spearman’s correlation index if they were normally or non-normally distributed. A stepwise selection procedure was used to identify the most relevant predictors for the multiple regression model with RER mag as the dependent variable. The inclusion criterion was set at a *p-*value of 0.10, while the exclusion criterion was fixed at 0.05. Kaplan–Meier survival with Log Rank testing and multiple Cox regression analysis was used to determine if the RER overshoot during recovery and other variables predict major adverse cardiac events (MACEs)/deaths and transplant/LVAD-free survival. Restricted mean survival time was defined as the difference between the survival probabilities, or absolute risk reduction, for a given time interval [12]. MACEs were defined as the composite of total cardiac death, myocardial infarction, stroke, hospitalization because of HF, and revascularization, including percutaneous coronary intervention. A statistical significance level of *p* ≤ 0.05 was used for all analyses.

## Results

### Patients characteristics

A total of 297 patients with HF were consecutively evaluated, excluding 54 because of a LVEF > 40%, 16 due to LVAD, and five because NYHA class IV. Of the remaining 222 patients, 24 with less than 4 min of monitored recovery and eight without clear termination of RER overshoot in the recorded recovery interval were excluded. The final study population included 190 patients with HFrEF and a control group of 103 apparently healthy subjects (Supplementary Fig. 1).

The characteristics of the study subjects are described in Table 1. A total of 85 patients had HFrEF due to post-ischemic dilated cardiomyopathy (45%), 58 had idiopathic dilated cardiomyopathy (31%), 19 with post-myocarditis dilated cardiomyopathy (10%), and 17 with advanced stage cardiomyopathies (9%). All these patients with advanced stage cardiomyopathies presented inherited aetiologies: six with genetically determined dilated cardiomyopathy, five patients with left ventricular non-compaction cardiomyopathy, three patients with obstructive hypertrophic cardiomyopathy, and three patients with arrhythmogenic cardiomyopathy. Eleven patients (5%) had other underlying causes such as acromegaly, scleroderma, chemotherapy, cocaine abuse, congenital heart disease, or valvular disease.

**Table 1:** Clinical characteristics of the study population

	HFrEF (*n* = 190)	Controls (*n* = 103)
Gender (men %)	158 (83%)	55 (53)
Age (years)	55.74 ± 13.04	52.39 ± 9.36
Height (cm)	171.65 ± 9.02	169.73 ± 8.99
Weight (kg)	79.93 ± 17.50	74.64 ± 16.44
BMI (kg/m^2^)	26.99 ± 4.88	25.83 ± 4.95
LVEF (%)	28.53 ± 6.45	-
Hb (g/dL)	13.13 ± 1.88	14.42 ± 1.29
Glycemia (mmol/L)	5.91 ± 2.15	5.15 ± 1.99
Total cholesterol (mmol/L)	3.76 ± 1.02	3.92 ± 1.18
HDL-cholesterol (mmol/L)	1.04 ± 0.32	1.03 ± 0.32
LDL-cholesterol (mmol/L)	2.39 ± 0.88	2.55 ± 0.94
Triglycerides (mmol/L)	1.30 ± 0.69	1.42 ± 0.90
BNP (ng/L)*	538 (237 – 1370)	-
Beta-blockers	168 (88%)	-
NYHA classes	Class I: 97 (51%)	-
	Class II: 58 (31%)	
	Class III: 35 (18%)	
	Class IV: 0 (0%)	

*BMI*, body mass index; *LVEF*, left ventricular ejection fraction; *Hb*, hemoglobin; *HDL*, high-density lipoprotein; *LDL*, low-density lipoprotein; *BNP*, brain natriuretic peptide; *NYHA*, New York Heart Association

^*^124 patients

### CPET: exercise and recovery kinetics

Patients included in the study exercised until perceived exhaustion without reporting any major adverse events. Patients with HFrEF showed significantly lower cardiorespiratory fitness and efficiency when compared to healthy subjects (Table 2).

**Table 2:** Cardiopulmonary exercise test parameters and recovery metrics of patients with HFrEF and healthy controls

	HFrEF (*n* = 190)	Controls (*n* = 103)	*p* value
Exercise parameters			
HR peak (bpm)	120.19 ± 24.46	156.48 ± 22.54	< 0.001
HR peak (% of predicted)	73.17 ± 14.05	93.28 ± 12.13	< 0.001
VO_2_ at VT (ml/min)	707.07 ± 253.88	1245.39 ± 461.46	< 0.001
VO_2_ at VT (ml/kg/min)	8.92 ± 2.95	16.95 ± 6.11	< 0.001
VO_2_ peak (ml/min)	1197.81 ± 453.82	2085.96 ± 598.80	< 0.001
VO_2_ peak (ml/kg/min)	15.02 ± 5.11	28.45 ± 7.84	< 0.001
VO_2_ peak (% of predicted)	56.29 ± 17.27	100.78 ± 27.42	< 0.001
O_2_/HR peak (ml/bpm)	10.45 ± 3.62	14.14 ± 3.23	< 0.001
O_2_/HR peak (% of predicted)	75.52 ± 22.93	100.67 ± 16.79	< 0.001
VCO_2_ peak (ml/min)	1479.10 ± 585.87	2572.16 ± 777.78	< 0.001
VE/VCO_2_ slope	34.42 ± 8.64	26.81 ± 3.64	< 0.001
OUES (ml/logL)	1270.47 ± 499.60	1965.80 ± 623.35	< 0.001
VO_2_/work rate slope (ml/W)	7.94 ± 1.38	10.77 ± 1.93	< 0.001
PETCO_2_ rest (mmHg)	29.58 ± 4.10	33.79 ± 2.40	< 0.001
PETCO_2_ max (mmHg)	33.73 ± 5.16	41.43 ± 3.76	< 0.001
ΔPETCO_2_ (mmHg)	4.16 ± 2.63	7.64 ± 2.13	< 0.001
RER peak	1.27 ± 0.14	1.25 ± 0.09	0.903
Recovery parameters^†^			
RER max	1.56 ± 0.23	1.65 ± 0.20	0.001
RER mag (%)	24.81 ± 14.47	31.42 ± 12.99	< 0.001
Time to RER max (s)	132.17 ± 44.94	111.68 ± 91.00	0.570
RER slope	16.92 ± 14.53	21.19 ± 15.98	0.037
PETO_2_ mag (%)	4.16 ± 3.80	8.03 ± 4.22	< 0.001
VE/VO_2_ mag (%)	32.23 ± 24.90	53.40 ± 28.50	< 0.001

Data are expressed as a mean ± the standard deviation. *HR*, heart rate; *VO*_*2*_, oxygen uptake; *VT*, first ventilatory threshold; *VE/VCO*_*2*_* slope*, minute ventilation/carbon dioxide production slope; *VCO*_*2*_, carbon dioxide production; *OUES*, oxygen uptake efficiency slope; *PETCO*_*2*_, partial pressure end-tidal carbon dioxide; *ΔPETCO*_*2*_, difference between rest and maximum value during exercise of PETCO_2_; *RER*, respiratory exchange ratio; *RER mag*, magnitude of the RER overshoot; *RER slope*, linear regression slope of the RER increase after the end of exercise; *PETO*_*2*_* mag*, magnitude of the partial pressure end-tidal oxygen overshoot; *VE/VO*_*2*_* mag*, magnitude of the oxygen equivalent overshoot

^†^157 patients presented a RER overshoot while 33 did not; 106 patients showed a PETO_2_ overshoot and 103 a VE/VO_2_ overshoot during the monitored recovery phase

During the recovery phase, patients with HFrEF presented a mean RER max of 1.56 ± 0.23 within 132.17 ± 44.94 s of recovery, leading to a magnitude of the RER overshoot of 24.81 ± 14.47% (RER slope 16.92 ± 14.53). Although there was no difference in RER peak during exercise between patients with HFrEF and healthy subjects (1.27 ± 0.14 vs 1.25 ± 0.09, *p* = 0.903), RER max was significantly lower in patients with HFrEF with similar time to RER max during recovery. Indeed, RER mag was found significantly reduced in the patients with HFrEF compared to healthy subjects (*p* < 0.001). Similarly, the extent of the VE/VO_2_ and PETO_2_ overshoot was significantly lower in HFrEF (both *p* < 0.001).

Correlations were assessed between the recovery metrics and the other prognostically important CPET parameters (Supplementary Table 1). Although the RER peak and the time to reach RER max showed few and weak correlations with the main parameters of cardiorespiratory fitness and efficiency, RER max, RER mag, and RER slope were found to be significantly correlated with most of these indices. At multiple linear regression, only VO_2_ peak and PETCO_2_ max remained independently associated with RER mag (Table 3).

**Table 3:** Multiple linear regression for RER mag

Predictors	Beta	95% confidence interval	*p* value
PETCO_2_ max (mmHg)	1.012	0.544–1.481	< 0.001
VO_2_ peak (ml/kg/min)	0.657	0.195–1.118	0.006

*R*^2^ / *R*^2^ adjusted = 0.259 / 0.249

*PETCO*_*2*_* max*, maximum value during exercise of partial pressure end-tidal carbon dioxide; *VO2*, oxygen uptake; *RER mag*, magnitude of the RER overshoot

### Subgroup analyses

Patients were categorized based on ventilatory and Weber classes, comparing RER recovery parameters. In HFrEF, RER max, RER mag, and RER slope were significantly higher in patients of lower ventilatory classes compared with those of higher ventilatory class (*p* < 0.001). Regarding aerobic capacity, RER mag and RER slope were significantly higher in the patients of Weber class A or B when compared with patients of Weber class C or D (*p* < 0.001; Supplementary Fig. 2; Supplementary Table 2).

Twenty-one patients presented EOV, showing lower aerobic capacity and cardiorespiratory efficiency as well as a reduced RER overshoot when compared to patients without EOV (RER mag 14.8 ± 8.6% vs 24.9 ± 14.5%).

Thirty-three patients (17.4% of HFrEF) did not show an increase of RER during the recovery phase, while a RER overshoot was present in all healthy subjects. Moreover, patients without RER overshoot presented lower maximal and submaximal aerobic capacity as well as cardiorespiratory efficiency compared to patients with a growth of the RER during recovery (Table 4).

**Table 4:** Presence or absence of RER overshoot. Clinical characteristics and cardiopulmonary exercise test parameters of the subgroups; patients with and without RER overshoot were compared

	No RER overshoot (*n* = 33)	RER overshoot (*n* = 157)	*p*
Gender (men %)	27 (82%)	131 (83%)	0.821
Age (years)	56.45 ± 13.30	55.59 ± 13.02	0.631
BMI (kg/m^2^)	26.04 ± 4.62	27.19 ± 4.93	0.274
LVEF (%)	26.52 ± 7.05	28.98 ± 6.26	0.046
Beta-blockers (%)	28 (85%)	140 (89%)	0.548
HR peak (bpm)	115.48 ± 27.02	121.18 ± 23.86	0.232
VO_2_ at VT (ml/kg/min)	7.15 ± 2.33	9.30 ± 2.93	< 0.001
VO_2_ peak (ml/kg/min)	11.04 ± 3.08	15.85 ± 5.06	< 0.001
VO_2_ peak (%)	42.24 ± 14.70	59.24 ± 16.33	< 0.001
O_2_/HR peak (ml/bpm)	7.90 ± 2.77	10.98 ± 3.56	< 0.001
VCO_2_ peak (ml/min)	1030.76 ± 335.59	1573.34 ± 584.20	< 0.001
VE/VCO_2_ slope	41.49 ± 8.66	32.93 ± 7.89	< 0.001
OUES (ml/logL)	868.98 ± 371.47	1354.86 ± 482.52	< 0.001
VO_2_/work rate slope (mL/W)	6.91 ± 1.59	8.16 ± 1.22	< 0.001
RER peak	1.33 ± 0.18	1.25 ± 0.13	0.027
PETCO_2_ rest (mmHg)	27.22 ± 3.91	30.07 ± 3.97	< 0.001
PETCO_2_ max (mmHg)	29.97 ± 0.76	34.53 ± 0.40	0.001
ΔPETCO_2_ (mmHg)	2.75 ± 1.83	4.45 ± 2.69	0.001

Data are expressed as a mean ± the standard deviation. *BMI*, body mass index; *LVEF*, left ventricular ejection fraction; *HR*, heart rate; *VO*_*2*_, oxygen uptake; *VT*, first ventilatory threshold; *VE/VCO*_*2*_* slope*, minute ventilation/carbon dioxide production slope; *VCO*_*2*_, carbon dioxide production; *OUES*, oxygen uptake efficiency slope; *RER*, respiratory exchange ratio; *PETCO*_*2*_, partial pressure end-tidal carbon dioxide; *ΔPETCO*_*2*_, difference between rest and maximum value during exercise of PETCO_2_

### Prognostic value of RER recovery kinetics in HFrEF

The mean follow-up time was 2.51 ± 1.17 years; 26 patients died, 24 underwent cardiac transplantation, while 13 additional patients underwent LVAD placement during this period. Sixty-four patients presented one or more MACEs: 36 hospitalizations because of HF, 16 hospitalizations for cardiac arrest or appropriate ICD discharge, six myocardial infarctions, four sudden cardiac deaths, two cardiogenic shocks, and one stroke.

**Fig. 2:**
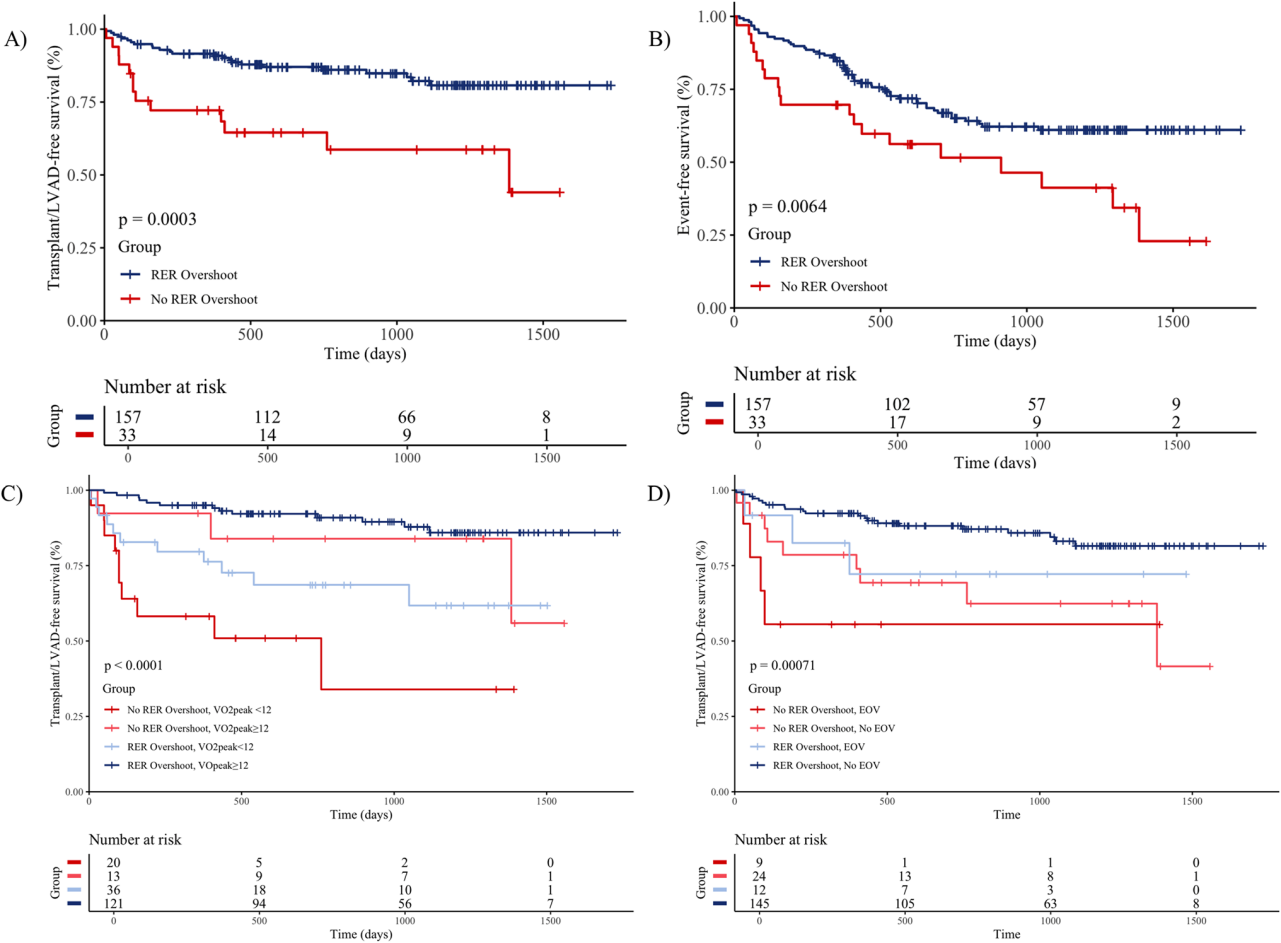
RER overshoot as prognostic indicator in patients with HFrEF. **A** Kaplan–Meier transplant/LVAD-free and **B** events (MACEs and deaths)-free survival curves for patients with HFrEF divided by the presence or absence of a RER overshoot during the recovery phase. **C** Kaplan–Meier transplant/LVAD-free survival curves stratified by VO_2_ peak and RER overshoot. **D** Kaplan–Meier transplant/LVAD-free survival curves stratified by the presence of EOV and RER overshoot. RER, respiratory exchange ratio; HFrEF, heart failure with reduced ejection fraction; LVAD, left ventricular assist device; MACE, major adverse cardiac events; VO_2_, oxygen uptake; EOV, exercise oscillatory ventilation

The absence of RER overshoot was associated with worse transplant/LVAD- and events-free survival in univariate Cox regression. In multiple Cox regression analysis, adjusting for possible cofounders, VO_2_ peak resulted as the only determinant in predicting transplant/LVAD-free survival (HR 0.883 every ml/kg/min, 95% CI 0.791–0.985, *p* = 0.025; Table 5), while VO_2_ peak and PETCO_2_ max as the determinants in predicting events-free survival (HR 0.921 every ml/kg/min, 95% CI 0.857–0.990, *p* = 0.026; HR 0.917 every mmHg, 95% CI 0.847–0.993, *p* = 0.033; respectively; Table 6). Kaplan–Meier curves for transplant/LVAD-free survival time stratified by the presence or absence of RER overshoot are shown in Fig. 2A; those patients with HFrEF not having a RER overshoot revealed poorer long-term outcomes (*p* < 0.001). The difference between the transplant/LVAD-free survival time between patients with and without RER overshoot was 364.99 days (95% CI 112.05–615.94, *p* = 0.005) during the monitored follow-up. Kaplan–Meier curves for events-free survival time and its association with the presence or absence of RER overshoot are shown in Fig. 2B; those patients with HFrEF not having a RER overshoot showed again poorer long-term outcomes (*p* = 0.006). The difference between the events-free survival time between patients with and without RER overshoot was 310.67 days (95% CI 65.19–556.14, *p* = 0.013), within the follow-up period.

**Table 5:** Multiple Cox regression analysis predicting transplant/LVAD-free survival

*Predictors*	*HR*	*95% CI*	*p*	*HR*	*95% CI*	*p*	*HR*	*95% CI*	*p*
No RER overshoot	3.255	1.654 – 6.407	**0.001**	3.087	1.537–6.201	**0.002**	1.649	0.765–3.552	0.201
Age (years)				1.003	0.976–1.032	0.808	0.993	0.965–1.022	0.628
Gender (male)				2.590	0.772–8.690	0.123	2.788	0.814–9.547	0.103
LVEF (%)				0.973	0.924–1.026	0.312	1.009	0.952–1.069	0.767
VO_2_ peak (for every 1 ml/kg/min)							0.883	0.791–0.985	**0.025**
VE/VCO_2_ slope (for every 1 increase)							1.035	0.979–1.093	0.224
PETCO_2_ max (for every 1 mmHg)							1.015	0.912–1.130	0.780

*RER*, respiratory exchange ratio; *LVEF*, left ventricular assist device; *VO*_*2*_, oxygen uptake; *VE/VCO*_*2*_* slope*, minute ventilation/carbon dioxide production slope; *PETCO*_*2*_* max*, maximum value during exercise of partial pressure end-tidal carbon dioxide

Bold values denote statistical significance at the *p* < 0.05 level

**Table 6:** Multiple Cox regression analysis predicting events-free survival

*Predictors*	*HR*	*95% CI*	*p*	*HR*	*95% CI*	*p*	*HR*	*95% CI*	*p*
No RER overshoot	2.040	1.209–3.443	**0.008**	1.815	1.059–3.109	**0.030**	1.268	0.711–2.261	0.422
Age (years)				1.009	0.990–1.028	0.352	1.000	0.980–1.020	0.989
Gender (male)				0.986	0.520–1.870	0.965	0.996	0.519–1.914	0.991
LVEF (%)				0.969	0.934–1.005	0.089	0.985	0.947–1.024	0.440
VO_2_ peak (for every 1 ml/kg/min)							0.921	0.857–0.990	**0.026**
VE/VCO_2_ slope (for every 1 increase)							0.978	0.935–1.022	0.319
PETCO_2_ max (for every 1 mmHg)							0.917	0.847–0.993	**0.033**

*RER*, respiratory exchange ratio; *LVEF*, left ventricular assist device; *VO*_*2*_, oxygen uptake; *VE/VCO*_*2*_* slope*, minute ventilation/carbon dioxide production slope; *PETCO*_*2*_* max*, maximum value during exercise of partial pressure end-tidal carbon dioxide

Bold values denote statistical significance at the *p* < 0.05 level

Among patients with severely reduced VO_2_ peak (< 12 ml/kg/min; *n* = 56), those not showing a RER overshoot during recovery were 20 (36%). This subgroup showed worse transplant/LVAD-free survival compared to those with reduced VO_2_ peak and determinable RER overshoot (Fig. 2C and Supplementary Table 3). Furthermore, among the 21 patients with EOV, nine showed no RER overshoot (43%). This subgroup also presented a worse transplant/LVAD-free survival compared to patients with EOV and RER overshoot (Fig. 2D and Supplementary Table 4).

## Discussion

While there is a wide body of literature investigating the gas exchange response to exercise in several populations, the clinical value of the recovery phase analysis is still limited [13]. The aim of the present study was to evaluate the recovery of respiratory gas indices after maximal CPET, with particular focus on the RER overshoot, in a population with HFrEF.

### Exercise recovery in HFrEF and subgroup analyses

The present study was the first to provide RER recovery parameters in a population of HFrEF.

Patients with HFrEF presented lower cardiorespiratory fitness and efficiency, associated with an attenuated recovery of overshoot parameters compared to healthy subjects. Indeed, although RER peak was similar between these groups, RER max and consequently RER mag were significantly lower in patients with HFrEF. Instead, time to RER max was comparable between the groups, with a RER slope appearing higher in controls. Similar behavior has already been described by Takayanagi et al. [6], while our study confirmed this phenomenon in a larger sample, showing how the time to RER max was similar between groups. Indeed, the overshoot occurs in the same time interval but on a smaller scale even in a population with HFrEF. Moreover, the respective direct and indirect correlations of RER mag with VO_2_ peak and VE/VCO_2_ slope were in line with those previously evaluated in patients with HFrEF [6]. The multiple regression analysis further suggests that the RER overshoot is determined by the severity of the disease and, consequently, by VO_2_ peak and PETCO_2_ during exercise. Thus, it was supposed that the magnitudes of the overshoots of respiratory gas indices are strictly related to cardiopulmonary function and responses during exercise more than to resting cardiac function.

To assess the potential clinical value of the RER overshoot evaluation, HFrEF patients were divided into subgroups based on functional classifications used in the prognostic risk stratification of these patients [14]. RER recovery parameters were altered in those patients belonging to the worse prognostic classes in terms of ventilatory efficiency (ventilatory class III and IV) and aerobic capacity (Weber class C and D), which is in line with what has been previously reported in kidney transplant recipients and patients with congenital heart disease [15, 16]. Furthermore, a vigorous RER overshoot, both in terms of intensity and speed with which it is achieved, seems to be a simple qualitative index of better cardiorespiratory performance and was associated with classes having a better prognosis in patients with HFrEF.

### No overshoot

**Fig. 3:**
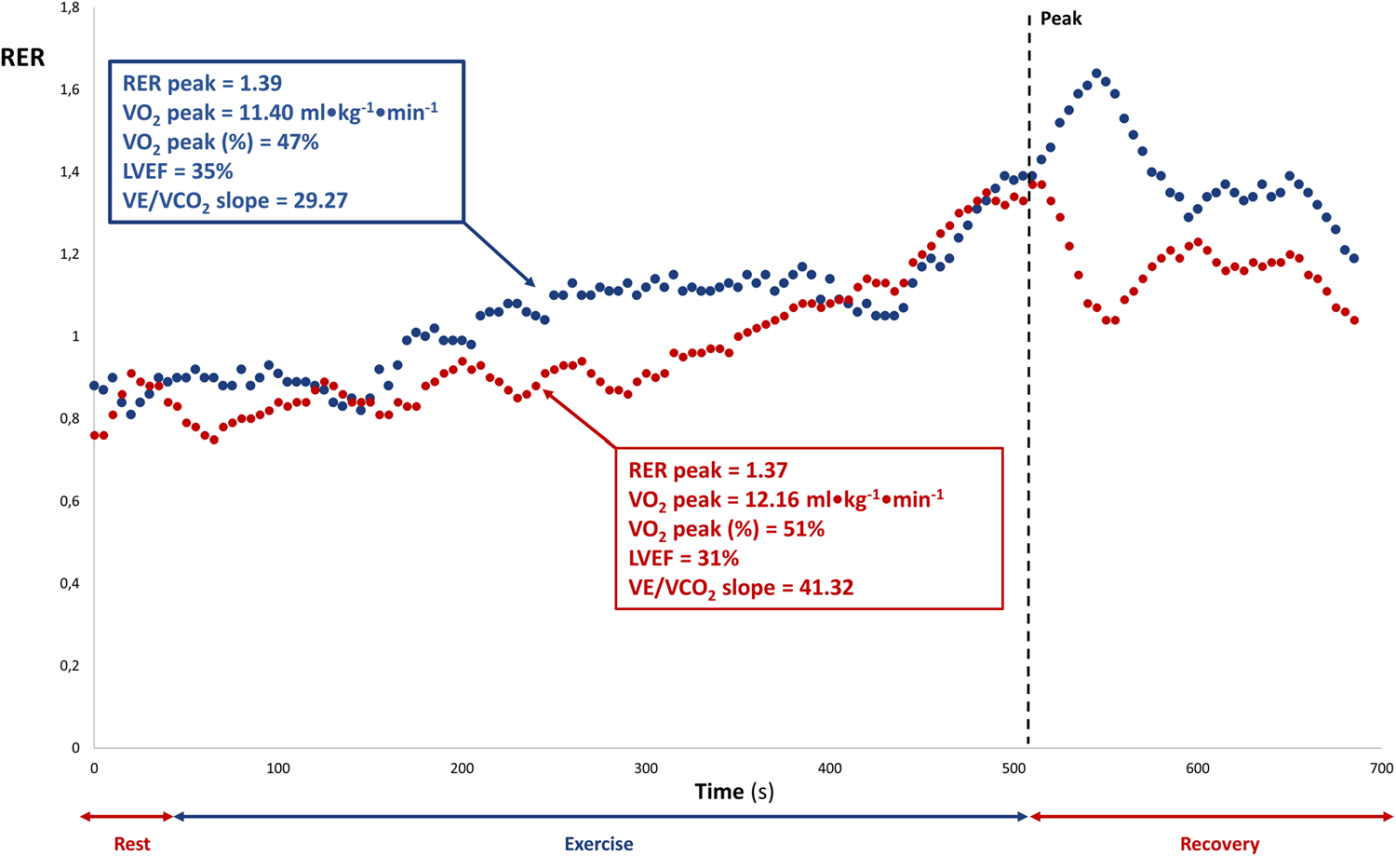
RER overshoot in clinical practice. An example of two patients with HFrEF presenting the same exercise time with similar RER peak, VO_2_ peak, and LVEF. The patient presenting a RER overshoot (blue) had a VE/VCO_2_ slope of 29.27, while the patient with no RER overshoot (red) showed a VE/VCO_2_ slope of 41.32, suggesting significantly different cardiorespiratory efficiency. RER, respiratory exchange ratio; HFrEF, heart failure with reduced ejection fraction; VO_2_, oxygen uptake; LVAD, left ventricular ejection fraction; VE/VCO_2_ slope, minute ventilation/carbon dioxide production slope

A small number of patients (17%) reported no RER overshoot during the recovery phase, a phenomenon not yet described in previous works. Interestingly, this subgroup of patients displayed significant cardiorespiratory impairments compared to patients with HFrEF presenting a RER overshoot, despite a higher RER peak. More specifically, patients without RER overshoot belong to a higher ventilatory class (VE/VCO_2_ slope 41.49 ± 8.66, class III vs 32.93 ± 7.89, class II) and a worse Weber class (VO_2_ peak 11.04 ± 3.08 ml/kg/min, class C/D vs 15.85 ± 5.06 ml/kg/min, class B/C). This functional discrepancy regarding cardiorespiratory efficiency and maximal/submaximal aerobic capacity suggests that patients with HFrEF who do not present a RER overshoot are at risk of worse clinical outcomes. This new finding may be useful for clinical purposes since patients with similar functional capacity, exercise tolerance, and resting LVEF may still have different cardiorespiratory responses during exercise and therefore different prognostic outcomes (Fig. 3).

The RER overshoot is not the first recovery parameter that can offer important information regarding patients’ prognoses. Slow VO_2_ kinetics and HR recovery immediately after exercise have been shown to be associated with disease severity [17]. This study also investigated the association of recovery parameters with hard clinical endpoints such as MACEs, deaths, cardiac transplantation, and/or LVAD placement, showing that the presence of a RER overshoot resulted as a good prognostic marker. Indeed, the absence of a RER overshoot tripled the risk of transplant and doubled the risk of fatal and non-fatal MACE in the monitored period. Despite this, in the Cox multiple linear regression, VO_2_ peak remains the sole determinant as a significant predictor for transplant/LVAD-free survival and is associated with the maximal PETCO_2_ during exercise for events-free survival. This should not be surprising since VO_2_ peak is recognized as the best prognostic marker in patients with HFrEF, but its association with the presence or absence of RER overshoot allows an improved multiparametric prognostic risk stratification. Indeed, in high-risk patients with severely reduced aerobic capacity and/or EOV, patients without RER overshoot presented a lower events- and transplant/LVAD-free survival. Therefore, the novelty and possible application of the RER overshoot lies in the ability to identify a subgroup of HFrEF patients with lower functional impairment and a better prognosis, especially in the most severely compromised patients, thus possibly influencing clinical considerations including the timing of heart transplantation or LVAD implantation. It is necessary to specify that the presence or absence of RER overshoot is a dichotomous parameter, while the remaining associated variables included in the model as VO_2_ peak and PETCO_2_ max are quantitative parameters. Thus, a future quantitative analysis could be made with the RER mag, also in absence of a RER overshoot, by giving this parameter a negative value.

### Gas exchange mechanisms explaining gas indices overshoot during recovery phase

VO_2_, VCO_2_, and VE typically return to their resting levels quite rapidly during recovery from maximal exercise. However, compared to VO_2_, the recovery of VCO_2_ and VE is generally delayed by the amount of CO_2_ stored in the body that needs to be removed. The overshoot phenomena of RER, but also of VE/VO_2_, are therefore the results from delayed recoveries of VCO_2_ and VE versus the relatively rapid recovery of VO_2_ [7].

Since patients with HFrEF presented lower recovery overshoot parameters compared to healthy subjects, it was suggested that this behavior can be attributed to the slower recovery of VO_2_ [18]. Different studies proposed possible gas exchange CPET variables to monitor VO_2_ kinetics during the recovery phase, such as time measurements, linear slope, and relative reduction in an established period post-test [19–22]. The most used parameter is probably the time constant (tau, τ), determined by a mono-exponential function fitted from the beginning to the end of the recovery period, that, despite a good validity and reproducibility [23], showed less predictive power than measurements closer related to peak exercise [20]. Another time parameter recently investigated is the VO_2_ recovery delay [24], which has been suggested as a non-invasive signal of an impaired adaptation of cardiac output during exercise [17, 19, 21] and was proven to be an independent predictor of cardiac transplant-free survival [24, 25].

Despite this, in clinical practice, it appears that a clear VO_2_ delay is easily recognized only when an overshoot phenomenon of VO_2_ is present during recovery. In our cohort, seven patients presented an identifiable VO_2_ overshoot during the recovery phase with only one of them showing a RER overshoot. Moreover, VO_2_ delay is not always easy to determine and occurs within the very first seconds of the recovery phase, which questions its direct relationship with other overshoot parameters and makes it clinically less feasible [24]. On the other hand, the time to RER max was slightly over 2 min in our study, making the RER overshoot an easily identifiable and immediately understandable secondary phenomenon, which in our opinion only partly reflects the VO_2_ delay during the early recovery phase. A recent study by Fortin et al. proposed the difference between VO_2_ peak and the VO_2_ measured at 2 min of recovery as a strong prognostic marker of death, heart transplantation, and LVAD implantation in severe HFrEF [22]. This proposed parameter, although reflecting only VO_2_ recovery kinetics, seems to be closer related with the results of our study as the timing of the phenomenon and its clinical relevance are comparable.

**Fig. 4:**
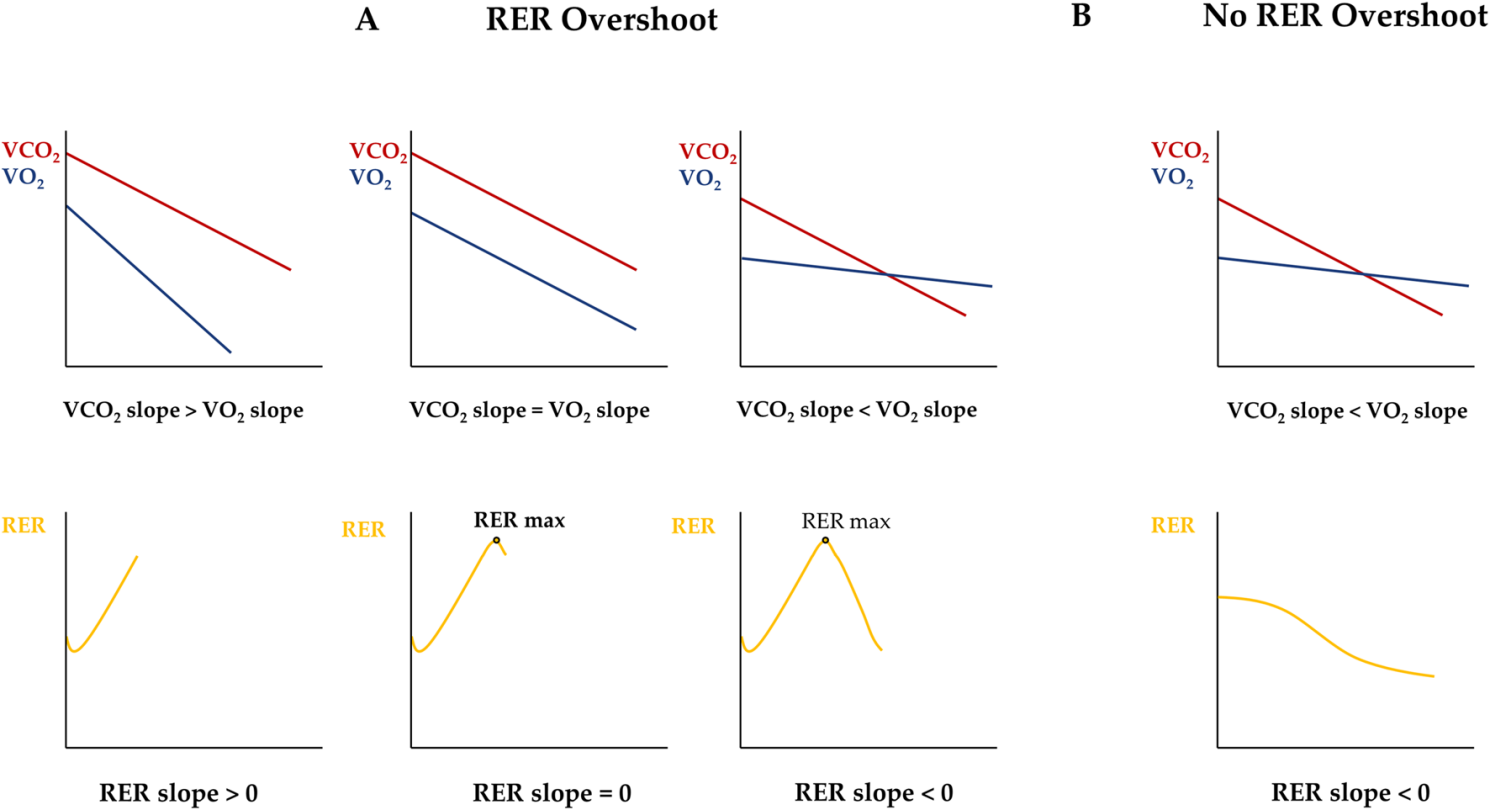
Pathophysiological mechanisms of the RER overshoot. **A** During the first phase of increase, the rate of decline of VCO_2_, with the respective recovery slope, is slower than that of VO_2_ (VCO_2_ slope > VO_2_ slope). When the VCO_2_ and VO_2_ slopes are equivalent, then the RER max is reached and the RER growth stops. Thereafter, the VO_2_ slope exceeds the VCO_2_ slope, and this results in a decrease of the RER. **B** When RER overshoot is not present, the VO_2_ slope is lower than the VCO_2_ slope from the beginning of the recovery phase. RER, respiratory exchange ratio; VCO_2_, carbon dioxide consumption; VO_2_, oxygen uptake

The reasons for the lack of RER increase during the recovery phase are not known. As the RER is the ratio between VCO_2_ and VO_2_, the rate at which these parameters return to baseline values during the recovery phase determines whether overshoot occurs. Normally, both parameters fall immediately at the end of the exercise phase, and the trend of RER is determined by the descent rate. In fact, usually VO_2_ falls with a faster rate than VCO_2_, repaying the oxygen deficit established in the initial phase of exercise and determining the post-exercise RER growth phase, i.e., overshoot. This behavior continues until a point is reached where the two rates of descent are equivalent, which defines the maximum RER measured during CPET recovery, called RER max. Thereafter, the descent of VCO_2_ exceeds the rate of VO_2_ decline causing RER to fall until it reaches the resting values. In case a RER overshoot is not present, the VCO_2_ descent rate is immediately greater than that of VO_2_ (Fig. 4). Interestingly, patients without overshoot had a higher RER peak than patients with overshoot. However, different maximal intensities/efforts reached during the test seem not to influence VO_2_ recovery kinetics [26]. Therefore, future studies should more investigate VCO_2_ recovery kinetics with a specific focus on PETCO_2_ considering its possible influence on the RER overshoot phenomenon. PETCO_2_ is a known ventilatory marker that also reflects impaired cardiac output during exercise, and its strong prognostic value has been demonstrated for patients with HFrEF [1, 27]. PETCO_2_ depends on exercise intensity (peripheral CO_2_ production) and cardiorespiratory efficiency. Therefore, low values reached during exercise, as identified in many patients with HFrEF, could lead to a VCO_2_ recovery delay, thus influencing the reduced or even failed RER growth during the first recovery phase.

Although the examination of gas exchange recovery kinetics variables may offer clinically relevant information, this approach has never become clinical practice, also because of non-feasible and time-consuming evaluations. A dichotomous but also quantitative parameter such as the presence of RER overshoot and its magnitude seems more easily adoptable for recovery kinetics because immediately identifiable, with good prognostic value and considering both VO_2_ and VCO_2_ kinetics.

Despite the absolute and primary relevance of VO_2_ peak in stratifying disease severity and prognostic risk, a simplified analysis of VO_2_ and VCO_2_ kinetics through RER behavior during the recovery phase from maximal exercise seems a promising step forward in CPET interpretation. A further understanding of complex pathways implicated in impaired respiratory gas exchange kinetics during recovery may help to improve diagnostic sensitivity and clinical decision-making in patients with HFrEF and beyond [15].

### Study limitations

This was a retrospective study, investigating the behavior of some recovery indices among CPET evaluations made for clinical purposes. For this reason, RER overshoot was not assessed according to a specified control group, consisting in subjects attending our outpatient clinic for a functional evaluation with maximal CPET with no history of structural or functional heart disease.

The study’s sample size and follow-up history are limited to January 2018, when a dedicated CPET recovery protocol was created. Finally, the analysis of subgroups with higher impairment and worse prognosis resulted in a further reduction in sample size, diminishing the statistical strength of the conclusions that could be obtained from the study. Consequently, the clinical relevance of the proposed RER overshoot metrics should be confirmed in larger prospective studies, including HF with preserved ejection fraction, and patients with different functional limitations to exercise.

## Conclusions

Overshoots of some respiratory gas indices are commonly observed during the exercise recovery phase after maximal CPET. These phenomena are attenuated in patients with HFrEF compared to controls and RER recovery parameters correlated with prognostically relevant CPET indices of cardiorespiratory fitness and efficiency in HFrEF. Interestingly, patients lacking a RER overshoot presented significant cardiorespiratory impairment compared to patients with RER overshoot showing worse events-, transplant- and LVAD-free survival. RER overshoot represents a new index to monitor gas exchange kinetics during the recovery phase, which may help clinicians to interpret patients’ functional impairment with crucial clinical decision-making drawbacks.

## Supplementary Information

Below is the link to the electronic supplementary material.

Supplementary file1 (DOCX 155 KB)

## Data Availability

The data presented in this study are available on request from the corresponding author.
