# Publisher Correction: Respiratory exchange ratio overshoot during exercise recovery: a promising prognostic marker in HfrEF

**DOI:** 10.1007/s00392-024-02416-3

**Published:** 2024-02-21

**Authors:** Marco Vecchiato, Daniel Neunhaeuserer, Emanuele Zanardo, Giulia Quinto, Francesca Battista, Andrea Aghi, Stefano Palermi, Luciano Babuin, Chiara Tessari, Marco Guazzi, Andrea Gasperetti, Andrea Ermolao

**Affiliations:** 1 Sports and Exercise Medicine Division, Department of Medicine, University of Padova, University Hospital of Padova, Via Giustiniani 2, 35128 Padua, Italy; 2 Fisioterapia Osteopatia Raimondi Di Giovanni e Daniele, Piazza Vittorio Veneto 1, Selvazzano Dentro, Padua, Italy; 3 Public Health Department, University of Naples Federico II, 80131 Naples, Italy; 4 Cardiology Unit, Department of Cardiac, Thoracic, Vascular Sciences and Public Health, University Hospital of Padova, Padua, Italy; 5 Cardiac Surgery Unit, Department of Cardiac, Thoracic, Vascular Sciences and Public Health, University Hospital of Padova, Padua, Italy; 6 Department of Biological Sciences, University of Milano School of Medicine, Milan, Italy; 7 Cardiology Division, San Paolo Hospital, Milan, Italy

**Publisher Correction: Clinical Research in Cardiology**

10.1007/s00392-024-02391-9

During production of this article, the affiliation of the first author was given incompletely.

Instead of:

“Sports and Exercise Medicine Division, Department of Medicine, University Hospital of Padova, Via Giustiniani 2, 35128 Padova, Italy”

there should have been:

“Sports and Exercise Medicine Division, Department of Medicine, University of Padova, University Hospital of Padova, Via Giustiniani 2, 35128 Padova, Italy”

The original article has been corrected.

The publisher apologises for the mistake.

