# Supplementary material for: Respiratory exchange ratio overshoot during exercise recovery: a promising prognostic marker in HFrEF
Source: Clin Res Cardiol. 2024 Feb 15;115(3):412–23. doi: 10.1007/s00392-024-02391-9 (PMC12894429; doi:10.1007/s00392-024-02391-9)

**Supplementary Table 1.** Associations between cardiorespiratory fitness/efficiency and RER overshoot metrics in HFrEF.

|  | **RER peak** | **RER max** | **RER mag** | **Time to RER max** | **RER slope** |
| --- | --- | --- | --- | --- | --- |
| **Age (years)** | - | - | - | - | -0.178** |
| **LVEF (%)** | - | 0.301** | 0.305** | 0.199* | 0.222** |
| **HR peak (bpm)** | - | 0.212* | 0.203* | - | 0.187* |
| **VO_2_ at VT (ml/kg/min)** | -0.225** | - | 0.307** | - | 0.288** |
| **VO_2_ peak (ml/kg/min)** | - | 0.304** | 0.430** | - | 0.386** |
| **O_2_/HR peak (ml/bpm)** | -0.146* | - | 0.350** | - | 0.327** |
| **VCO_2_ peak (ml/min)** | - | 0.480** | 0.474** | - | 0.448** |
| **VE/VCO_2_ Slope** | - | -0.419** | -0.484** | - | -0.447** |
| **OUES (ml/logL)** | -0.288** | - | 0.418** | - | 0.350** |
| **VO_2_/Watt Slope (ml/W)** | -0.391** | - | 0.310** | - | 0.346** |
| **RER peak** | / | 0.650* | - | - | - |
| **PETCO_2_ rest (mmHg)** | - | 0.235** | 0.306** | - | 0.301** |
| **PETCO_2_ max (mmHg)** | - | 0.387** | 0.503** | - | 0.460** |
| **ΔPETCO_2_ (mmHg)** | - | 0.371** | 0.425** | - | 0.355** |
| Correlations between CPET parameters during exercise and RER recovery overshoot parameters, expressed as Spearman’s rho for non-normally distributed data*.*  LVEF = left ventricular ejection fraction; HR = heart rate; VO_2_ = oxygen uptake; VT = first ventilatory threshold; VE/VCO_2_ slope = minute ventilation/carbon dioxide production slope; VCO_2_ = carbon dioxide production; OUES = oxygen uptake efficiency slope; RER = respiratory exchange ratio; RER mag = magnitude of the RER overshoot; RER slope = linear regression slope of the RER increase after the end of exercise; PETCO_2_ = partial pressure end-tidal carbon dioxide; ΔPETCO_2_ = difference between rest and maximum value during exercise of PETCO_2_.  * < 0.05; ** < 0.01. | | | | | |

**Supplementary Table 2.** The association of RER overshoot and Ventilatory/Weber classes.

| **All patients with  RER overshoot** (n = 157) | **Ventilatory Class I** VE/VCO_2_ slope  < 30  (n = 63) | **Ventilatory Class II** VE/VCO_2_ slope  30-35.9  (n = 54) | **Ventilatory Class III** VE/VCO_2_ slope  36-44.9  (n = 28) | **Ventilatory Class IV** VE/VCO_2_ slope  > 45  (n = 12) | **p value** |
| --- | --- | --- | --- | --- | --- |
| **RER max** | 1.68 (1.49 - 1.86) | 1.49 (1.38 - 1.63) | 1.41 (1.28 - 1.59) | 1.39 (1.32 - 1.56) | <.001 |
| **RER mag (%)** | 29.10 (22.2 - 44.7) | 20.05 (13.25 - 27.10) | 18.30 (9.73 - 25.58) | 9.55 (7.05 - 19.50) | <.001 |
| **Time to RER max (s)** | 122.0 (95.0 - 157.0) | 130.0 (114.3 - 167.5) | 136.5 (116.3 - 165.0) | 107.0 (75.3 - 167.0) | .292 |
| **RER slope** | 16.20 (11.44 - 33.33) | 12.45 (8.29 - 19.92) | 8.54 (4.62 - 15.15) | 6.75 (2.86 - 12.00) | <.001 |
| **All patients with  RER overshoot** (n = 157) | **Weber Class A** VO_2_ peak  > 20 ml/kg/min  (n = 32) | **Weber Class B** VO_2_ peak  16-20 ml/kg/min  (n = 37) | **Weber Class C** VO_2_ peak  10-16 ml/kg/min  (n = 76) | **Weber Class D** VO_2_ peak  < 10 ml/kg/min  (n = 12) | **p value** |
| **RER max** | 1.65 (1.45 - 1.82) | 1.59 (1.45 - 1.83) | 1.50 (1.36 - 1.65) | 1.43 (1.29 - 1.56) | .020 |
| **RER mag (%)** | 30.20 (23.96 - 43.63) | 23.30 (17.55 - 40.30) | 19.90 (11.40 - 26.45) | 16.25 (9.25 - 20.68) | <.001 |
| **Time to RER max (s)** | 112.5 (88.5 - 156.8) | 128.0 (108.5 - 182.5) | 130.0 (106.0 - 155.5) | 131.5 (81.0 - 174.5) | .618 |
| **RER slope** | 19.69 (12.75 - 32.80) | 13.63 (9.25 - 23.91) | 11.40 (6.31 - 16.36) | 8.26 (6.06 - 20.71) | .001 |

RER recovery parameters (RER max, RER mag, Time to RER max, and RER slope) in patients with HFrEF belonging to different ventilatory efficiency- and Weber- classes; only patients presenting a RER overshoot could be included in this table (n =157).

**Supplementary Table 3.** Post-hoc analysis of Kaplan-Meier transplant/LVAD-free survival curves grouped by VO_2_ peak and the presence of RER overshoot.

| ***Subgroup comparison*** | | | ***χ²*** | ***p-value*** | | |
| --- | --- | --- | --- | --- | --- | --- |
|  |  |  |  | ***Raw*** | ***Tukey-Kramer*** | |
| **No RER overshoot, VO_2_ peak < 12 ml/kg/min** | **No RER overshoot, VO_2_ peak ≥ 12 ml/kg/min** | 12.7846 | | 0.0003 | 0.0020 |  |
| **No RER overshoot, VO_2_ peak < 12 ml/kg/min** | **RER overshoot, VO_2_ peak < 12 ml/kg/min** | 12.0150 | | 0.0005 | 0.0030 |  |
| **No RER overshoot, VO_2_ peak < 12 ml/kg/min** | **RER overshoot, VO_2_ peak ≥ 12 ml/kg/min** | 31.2194 | | <0.0001 | <0.0001 |  |
| **No RER overshoot, VO_2_ peak ≥ 12 ml/kg/min** | **RER overshoot, VO_2_ peak < 12 ml/kg/min** | 3.8382 | | 0.0501 | 0.2036 |  |
| **No RER overshoot, VO_2_ peak ≥ 12 ml/kg/min** | **RER overshoot, VO_2_ peak ≥ 12 ml/kg/min** | 0.0867 | | 0.3632 | 0.7999 |  |
| **RER overshoot, VO_2_ peak < 12 ml/kg/min** | **RER overshoot, VO_2_ peak ≥ 12 ml/kg/min** | 16.1462 | | <0.0001 | 0.0003 |  |

RER = respiratory exchange ratio; VO_2_ = oxygen uptake.

**Supplementary Table 4.** Post-hoc analysis of Kaplan-Meier transplant/LVAD-free survival curves grouped by the presence of EOV and RER overshoot.

| ***Subgroup comparison*** | | | ***χ²*** | ***p-value*** | | |
| --- | --- | --- | --- | --- | --- | --- |
|  |  |  |  | ***Raw*** | ***Tukey-Kramer*** | |
| **No RER overshoot, EOV** | **No RER overshoot, no EOV** | 0.0634 | | 0.8012 | 0.9944 |  |
| **RER overshoot, EOV** | **RER overshoot, no EOV** | 10.5188 | | 0.0012 | 0.0065 |  |
| **No RER overshoot, no EOV** | **RER overshoot, EOV** | 1.3677 | | 0.2422 | 0.6462 |  |
| **No RER overshoot, EOV** | **RER overshoot, no EOV** | 17.9770 | | <0.0001 | 0.0001 |  |
| **No RER overshoot, no EOV** | **RER overshoot, no EOV** | 1.7116 | | 0.1908 | 0.5576 |  |
| **No RER overshoot, EOV** | **RER overshoot, EOV** | 9.3732 | | 0.0022 | 0.0118 |  |

EOV = exercise oscillatory ventilation.; RER = respiratory exchange ratio.

**Supplementary Figure 1: Study flow chart**

297 patients with HF were consecutively evaluated during the study period. 75 patients were excluded and 222 met the inclusion criteria. 32 eligible patients were further excluded because recovery protocol was not fully respected for technical issue or RER max was not clearly identifiable during recovery phase. The final study population included 190 patients with HFrEF and a control group of 103 apparently healthy subjects with no functional or structural heart disease.

HFrEF = heart failure with reduced ejection fraction; LVEF = left ventricular ejection fraction; LVAD = left ventricular assist device; NYHA = New York Heart Association.

**
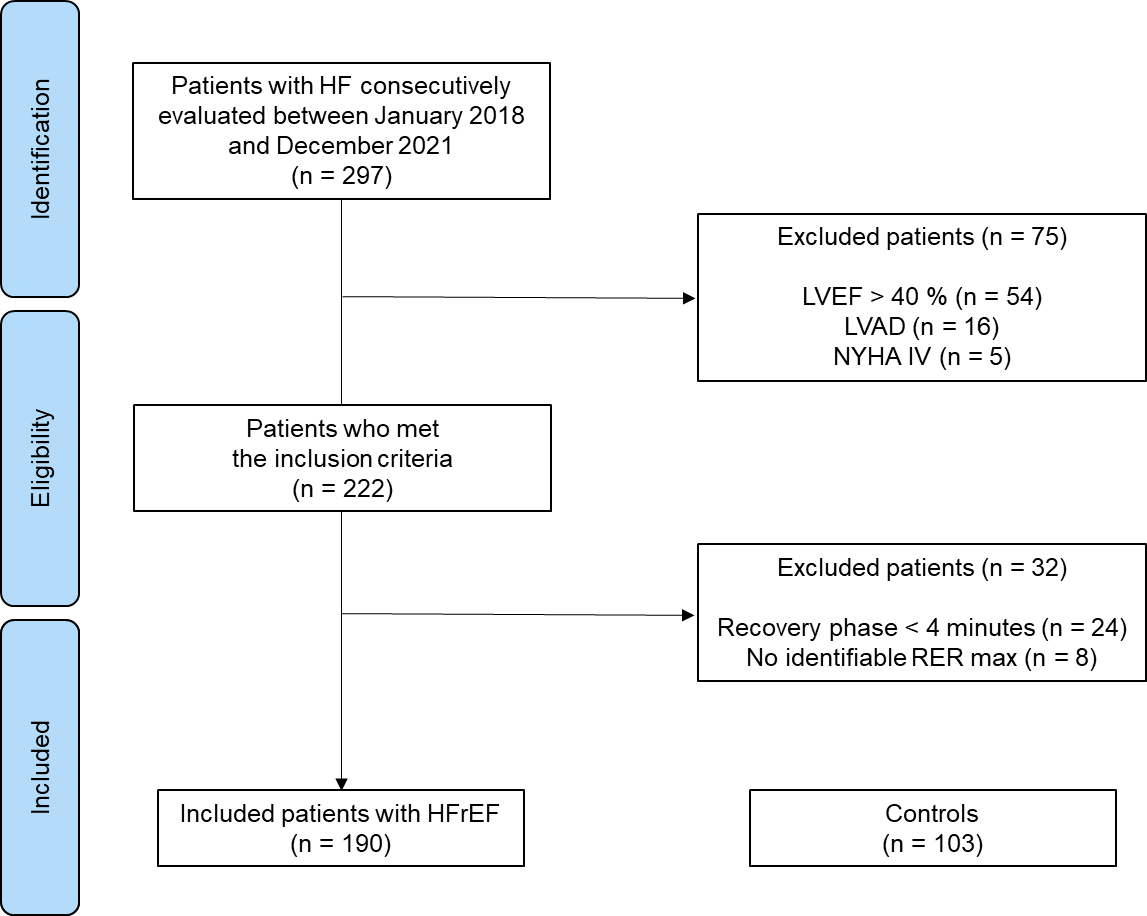
**

**Supplementary Figure 2: RER recovery parameters in patients with HFrEF stratified for ventilatory and Weber classification system**

RER recovery parameters (RER max, RER mag, Time to RER max, and RER slope) between patients belonging to different ventilatory efficiency classes (A, left column) and Weber fitness classes (B, right column).
RER = respiratory exchange ratio; HFrEF = heart failure with reduced ejection fraction; VC = Ventilatory class; WB = Weber class.

The number of * indicates the level of significance between subgroups:

* : vs VC I or WC A; ** : vs VC II or WC B; *** : vs VC III or WC C; **** : vs VC IV or WC D.


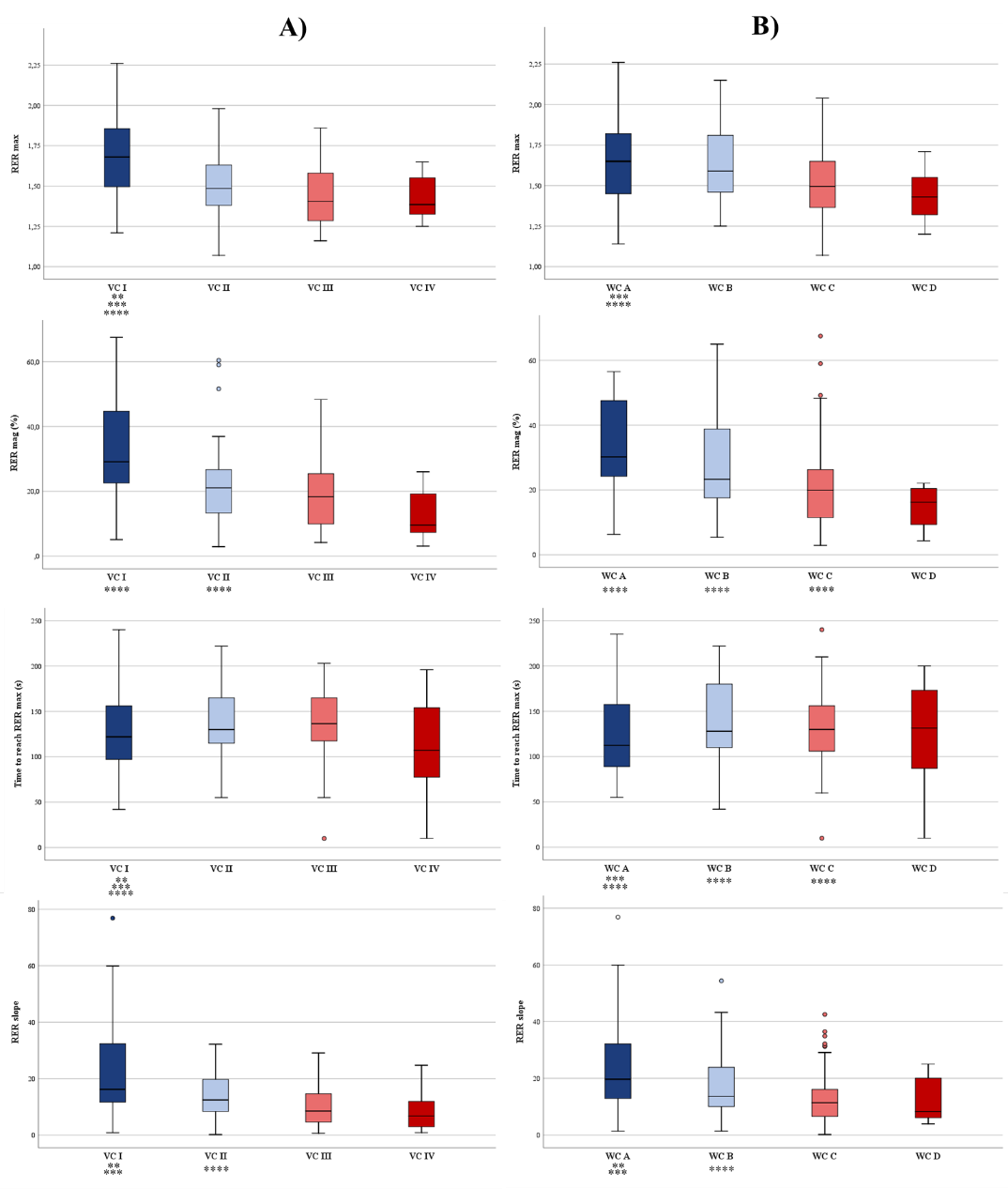

Supplement: Supplementary file 1 — Supplementary file1 (DOCX 155 KB) [file 392_2024_2391_MOESM1_ESM.docx]
